# Laryngeal Elevation Velocity and Aspiration in Acute Ischemic Stroke Patients

**DOI:** 10.1371/journal.pone.0162257

**Published:** 2016-09-01

**Authors:** Jing Zhang, Yun Zhou, Na Wei, Bo Yang, Anxin Wang, Hai Zhou, Xingquan Zhao, Yongjun Wang, Liping Liu, Melody Ouyoung, Brenda Villegas, Michael Groher

**Affiliations:** 1 Department of Neurology, Beijing Tiantan Hospital, Capital Medical University, Beijing, China; 2 China National Clinical Research Center for Neurological Diseases, Beijing, China; 3 Stroke Center, Beijing Institute for Brain Disorders, Beijing, China; 4 Beijing Key Laboratory of Translational Medicine for Cerebrovascular Disease, Beijing, China; 5 Department of Radiology, Beijing Tiantan Hospital, Capital Medical University, Beijing, China; 6 Department of Speech Pathology, Keck Hospital of University of Southern California, Los Angeles, California, United States of America; 7 Department of Otolaryngology-Head and Neck Surgery, University of Southern California, Los Angeles, California, United States of America; 8 Department of Communicative Disorders, University of Redlands, Redlands, California, United States of America; Chinese Academy of Sciences, CHINA

## Abstract

**Objectives:**

Aspiration after stroke has been associated with aspiration pneumonia, which contributes to increased mortality of stroke. Laryngeal elevation is a core mechanism for protection from aspiration. Few studies have explored the predictive value of laryngeal elevation velocity for aspiration after stroke. This study aimed to explore the ability of laryngeal elevation velocity to predict aspiration in patients with acute ischemic stroke.

**Methods:**

This was a prospective cohort study that included consecutive acute ischemic stroke patients treated at a teaching hospital during a 10-month period. Patients underwent magnetic resonance imaging (MRI) to confirm the diagnosis of acute ischemic stroke. Patients who were at risk of aspiration and could swallow 5 ml of diluted barium (40%, w/v) for a videofluoroscopic swallowing (VFS) study were included. The association between abnormal indices in the oral and pharyngeal phase of the VFS study and aspiration was examined using univariate analyses. These indices included the lip closure, tongue movement and control, laryngeal elevation velocity and range, the latency of pharyngeal swallowing, pharyngeal transit time (PTT), abnormal epiglottis tilt, residual barium in the pharynx, and the duration of upper esophageal sphincter (UES) opening. The laryngeal elevation velocity (%/s) was calculated as the range of laryngeal elevation (%) from the resting position to the maximum superior position or to the position where the laryngeal vestibule is fully closed divided by the corresponding duration of laryngeal elevation. The range of laryngeal elevation (%) was the percentage calculated as the distance between the resting laryngeal position and the maximum superior excursion position or position where the laryngeal vestibule is fully closed divided by the distance between the resting laryngeal position and the lowest edge of the mandible. A logistic regression analysis was used to determine the predictive value for aspiration secondary to reduced laryngeal elevation velocity after adjusting for the effects of other indices. Intrarater and interrater reliability were calculated using Pearson’s correlation coefficients.

**Results:**

Data from 89 patients were analyzed. This cohort included 71 males and 18 females with a mean age of 59.31±11.46 years. The mean time from stroke onset to the VFS study was 3 days (1–7). Twenty one (23%) patients aspirated while swallowing 5 ml of diluted barium (40%, w/v). Aspiration was associated with age, the velocity (%/s) of laryngeal elevation and duration, delayed pharyngeal phase, pharyngeal transit time, abnormal epiglottic tilt, and invalid laryngeal elevation before true swallowing, and duration of upper esophageal sphincter (UES) opening. After adjusting for the effects of the indices mentioned above, logistic regression analysis revealed that a reduced of laryngeal elevation velocity before vestibule closure was predictive of aspiration independently (OR, 0.993; 95% CI, 0.987–1.000).

**Discussions:**

Reduced laryngeal elevation velocity for laryngeal elevated to position where laryngeal vestibule is fully closed was an independent predictor of aspiration in patients with acute ischemic stroke. This may be related to a decreased contraction velocity of the muscles involved in hyolaryngeal elevation. Therapeutic methods aimed at improving laryngeal elevation velocity may decrease aspiration events and pneumonias after stroke.

## Introduction

More than 50% of stroke survivors may suffer from dysphagia [1]. Aspiration is one of the most feared complications of dysphagia after stroke [1]. since patients with aspiration are at an increased risk for the sequelae of airway obstruction or aspiration pneumonia[2]. Aspiration pneumonia is an infection of the lungs caused by aspirated bacteria [3]and remains the leading cause of death excluding the initial damage from the stroke itself.[3]Aspiration pneumonia accounts for third leading cause of death during the first month after stroke onset and approximately 34% of all stroke deaths thereafter [4]. The risk of developing pneumonia may be associated with the severity of aspiration after the stroke. Stroke patients with immune suppression and poor oral health are more susceptible to pneumonia if they aspirated, especially during the acute phase. Strategies to reduce aspiration could prevent pneumonia and improve outcomes in patients with acute stroke [5].

Therefore, a major aim of dysphagia assessment is to identify pathophysiological changes in swallowing that may cause aspiration after stroke [6]. The videofluoroscopic swallow study (VFS) assessment can demonstrate the presence and extent of aspiration and the potential aspiration mechanism during swallowing [7]. Identifying an abnormal change in the swallowing biomechanics could aid in identifying patients at risk of aspiration and decrease the incidence of aspiration pneumonia. Previous studies have identified several pathophysiological indices associated with aspiration including tongue strength, hyoid movement, bolus dwell time in the pharynx while the larynx remains open, respiratory rate, and respiration-swallow phasing [6]. Molfenter and his colleagues reported that the opening time of the upper esophageal sphincter (UES), time of laryngeal closure, stage transition duration and pharyngeal transit time were associated with development of aspiration [8]. Park et al. found that the initiation of laryngeal closure was associated with aspiration [9], while Power and her colleagues revealed that a model combining pharyngeal transit time, swallow response time and laryngeal closure duration could predict 73.11% of aspiration events [10].

Laryngeal elevation is a known important protective mechanism during swallowing [3]. This action contributes to moving the larynx out of the path of the bolus, as well as to the closure of the vestibule and opening of the UES [11]. A crucial event in the oropharyngeal phase is superior and anterior laryngeal excursion[12]. Abnormal laryngeal function may have close relationship with aspiration which could be indicated by the extent and speed of the superior and anterior excursion. It is important to use objective, instrumental techniques to measure hyolaryngeal function [13]. Previous studies showed that reduced laryngeal elevation was seen in dysphagia patients and associated with aspiration,[14]. Studies demonstrated reduced hyoid elevation associated with aspiration [15–17], which may be considered that reduced laryngeal elevation may associated with aspiration because the close relationship between laryngeal and hyoid which moved together as a complex[12].

However, laryngeal elevation velocity remains relatively unexplored [18]. Kahrilas and colleagues used two time parameters in a stepwise regression analysis to model a laryngeal penetration index. The two parameters were the duration between the glossopalatal junction opening and laryngeal vestibule closure (LVC) or the timing of the UES opening. Their model could explain 86% variation of the severity of laryngeal penetration among the neurogenic dysphagics. Their study concluded that delayed and slowed laryngeal elevation were important physiologic markers associated with penetration and aspiration [19]. However, these authors did not directly examine laryngeal elevation velocity or include a homogeneous group of stroke dysphagic patients. Our current study aims to measure the velocity of laryngeal elevation using frame-by-frame larynx-position tracking across a moving trajectory to identify its association with aspiration in stroke patients.

## Materials and Methods

### Ethics approval

The Institutional Review Board of the Beijing Tiantan Hospital approved this study.

### Subjects

This study was a prospective cohort study. This study had been conducted according to the principles expressed in the Declaration of Helsinki. The IRB (Institutional Review Board) of the Beijing Tiantan Hospital approved the study protocol. The written informed consent was obtained from the patient or their substitute decision makers. Patients were admitted to the stroke unit with acute ischemic stroke at a teaching hospital in Beijing, China during a 10-month period from July 2006 to April 2007. **Inclusion criteria**: The included patients had a diagnosis of stroke in accordance with the criteria of the World Health Organization. The time from stroke onset to enrollment in this study was less than 7 days. Patients were verified to have had an acute ischemic stroke by magnetic resonance imaging (MRI). A neurologist who worked as a speech- language pathologist in stroke unit (author 2)assessed the patients and determined the following: (1) if the patient had a history of coughing and choking while eating or drinking after the stroke; (2) a bedside swallow examination indicated possible aspiration, including reduced tongue movement, slowed initiation of pharyngeal swallow, reduced laryngeal elevation, coughing or a wet voice after swallowing; (3) patients with certain mental or/and physical status could complete the videofluoroscopic swallow study (VFS), and swallow 5 ml of diluted barium. **Exclusion criteria**: Patients with the following conditions were excluded: those who (1) could not undergo the MRI examination; (2) could not undergo the videofluoroscopic study or refused this examination; (3) were unable to maintain a sitting or standing posture and could not follow directions because of sensory aphasia or poor cognitive abilities; (4) had a history of structural disease affecting the mouth, pharynx or larynx, such as head and neck cancer, or tracheostomy, adenoidectomy, or neurodegenerative disease; (5) had an acute medical complication that prevented them from completing the swallowing assessment; and (6) could not complete an entire complete swallow, such as patients with severe Wallenberg syndrome.

### Videofluoroscopic swallowing study

A VFS was performed using a standardized recording protocol in the radiology suite [7]. A radiologist (author 7) was involved in all studies. The neurologist (author 1) performed the VFS study. Patients were placed in upright seated or standing position and consumed materials of varying textures (diluted, pudding-like, cookies) with barium and recorded in the lateral position and/or anterior-posterior position. Patients were first asked to dry swallow to further confirm whether they could completely swallow 5 ml or more of diluted, thick and solid barium without high risk of aspiration. Patients were asked to swallow 5 ml of diluted barium for 3 times if they could tolerate it. If patients aspirated, they will be asked to voluntarily cough and drained with certain posture. The liquid barium was diluted with water from barium sulfate (barium sulfate160%, w/v; pharmaceutical preparation room in Capital Medical University, Beijing, China) to 40%, w/v. Pudding-like barium was barium sulfate 160%w/v mixed with mushed banana and 1/4 cookie coated with barium was used as solid food.

The fluoroscopic studies were recorded on a digital Imagine workstation at 30 frames per second (Beijing Medex Corporation, Beijing, China). Images were viewed and analyzed frame by frame.

### Image analysis

**Fig 1:**
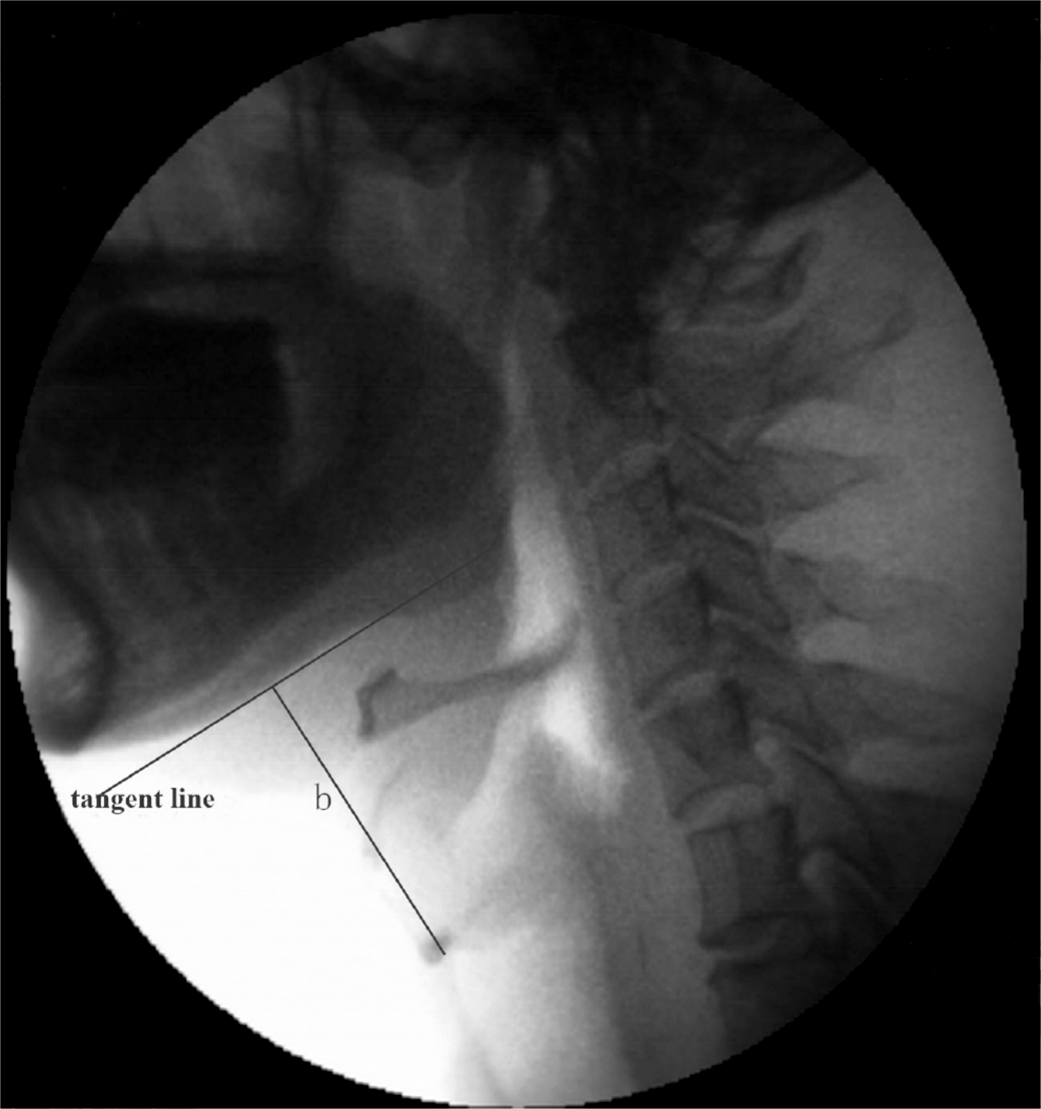
Lateral view of a videofluoroscopic image, showing the tangent line at the laryngeal rest position. The line b, perpendicular to the tangent line, illustrates the distance between the larynx and mandible.

A neurologist (author 1) who had swallowing assessment training experience analyzed the data frame by frame after the examination and again 2 weeks later. The neurologist (author 2) analyzed the data from 20 randomly-selected patients. Both of these two authors had been trained how to identify the resting position, maximal vertical elevation position and the position where the vestibule is fully closed. Also they were trained about how to measure the distance about the laryngeal elevation for 1 week. Only the frames involving the 5ml diluted barium swallows were analyzed. The specific aspects of oral phase and pharyngeal phase were noted and recorded. In oral phase, abnormal lip closure, decreased tongue movement and control were recorded. In pharyngeal phase, delay pharyngeal swallowing, duration of laryngeal vestibule closure (DLVC), laryngeal elevation range and time, presence of residue in pharyngeal (on valleculae, pyriform sinuses and/or post pharyngeal wall), opening time of UES, pharyngeal transit time (PTT), epiglottics tilt, invalid laryngeal elevation before true swallowing, penetration, silent aspiration and aspiration were recorded. The temporal parameters were converted from frames to seconds (s) by multiplying 1/30. Because the dysfunction of the pharyngeal phase may contribute to aspiration more than that of oral phase [4], this study focused mainly on the abnormal indices during the pharyngeal phase with the velocity of laryngeal elevation as the primary focus. To rule out the potential effects of other indices which may result in aspiration, indices with potential confounding effects analyzed in this study were also listed below:

1. Aspiration and penetration: Traditionally, aspiration was considered when any barium was seen traveling past the true vocal folds. A 8 point penetration and aspiration scale (PAS) was used to measure penetration and aspiration[20]. Patients were considered to aspiration with PAS score of greater than or equal to 5. Patients were considered to be a non-aspirator with PAS score of less than 5[21]. Based on its timing, aspiration was classified into three types: aspiration before, during and after swallowing. Aspiration before swallowing was defined as premature liquid spillage into the unprepared pharynx. Aspiration after swallowing was defined as the presence of excessive amounts of food residue in the pharynx after swallow and overflow into the trachea.
2. Range of larynx elevation (%): To minimize the effects of radiologic magnification and the differences in neck length among individuals, we calculated the percentage of laryngeal elevation as the range of the laryngeal elevation. Two indicators of the range of laryngeal elevation were calculated as follows.i. The percentage laryngeal elevation (%) during maximal elevation was defined as the vertical distance between the laryngeal resting position and the superior maximal excursion position divided by the distance between the laryngeal resting position and the lower ramus of the mandible. ii. The percentage laryngeal elevation (%) during full laryngeal closure was defined as the vertical distance between laryngeal resting position and its position where the larynx is fully closed divided by the distance between the laryngeal resting position and the lower ramus of the mandible. A few patients may accommodate the laryngeal position with a slight elevation different from the resting position, so that the resting position used for calculation was obtained from the frame of elevation burst. The distance between the laryngeal resting position and the lower ramus of the mandible was defined as the vertical distances (mm) (showed by line b in Fig 1) between the anterior corner of the air column of the trachea and the tangent line below the lower edge of mandible (Fig 1).
3. Duration of laryngeal elevation (s): Two indicators of the duration of laryngeal elevation were calculated as follows. i. Time required for laryngeal elevation from resting position to maximum superior elevation. ii. Time required for laryngeal elevation from resting position to its position where the laryngeal vestibule is fully closed. Videos of VFS were analyzed by neurologists frame to frame and the time was calculated as the number of the relevant frames divided by 30.
4. Velocity of laryngeal elevation (%/s): Two indicators of the velocity of laryngeal elevation (%/s) were calculated as follows. i. Velocity of laryngeal elevation to maximal superior position was defined as the range of laryngeal elevation to the maximal superior position (%) divided by the corresponding time. ii. Velocity of laryngeal elevation to full closure was defined as the range of laryngeal elevation to its position where the laryngeal vestibule was fully closed divided by the corresponding time.
5. Latency of the pharyngeal phase (s): This was defined as the delay between the onset of pharyngeal phase of swallowing and the elevation of laryngeal. The onset of pharyngeal phase was defined when the barium head reached the intersection between the lower edge of the mandibular ramus and tongue base.
6. Duration of opening of the UES (DOU) (s). This was defined as the time difference between the opening and full closure of the upper esophageal sphincter (UES) on lateral images.
7. Pharyngeal transition time (PTT) (s): The PTT was measured from the instant when the barium head reached the intersection between the lower edge of the mandibular ramus and tongue base to the instant when the barium tail passed the entrance into the esophagus.
8. Duration of laryngeal vestibule closure (DLVC) (s): The duration was defined as the time between the first and last frames of the LVC. LVC occurs by the elevation of the larynx against the underside of the epiglottis.
9. Barium residue in the pharynx: This included cumulative amount of barium residue on the valleculae, or pyriform sinuses other than any trace coating, or the coating of the pharyngeal wall that appeared after repeated swallowing.
10. Abnormal epiglottic tilt: normally, the epiglottis moves from its vertical resting position downward to seal the entrance into the laryngeal vestibule at the appropriate time. Abnormal epiglottic tilt was defined as insufficient, delayed or absent epiglottic tilt.
11. Invalid laryngeal elevation before true swallowing: This is defined as any swallowing attempts demonstrated as any extent elevation of laryngeal before a true or completely swallowing which did not reach maximal elevation position and did not result in any completely swallowing.

### Statistical analysis

Data of timing, distance, and velocity were presented as the means±standard deviation (SD) for normally distributed data and as the median with minimum/maximum for non-normally distributed data. Comparisons between groups were performed using the t-test or rank-sum test. Pearson correlation coefficients were calculated to assess the relationships among indices. The indices with a significant difference between groups according to the univariate analysis were analyzed with a logistic regression to identify the effect of the velocity of laryngeal elevation on aspiration. Parameters were subsequently entered into the multiple regression analysis if they achieved a 0.05 level of significance. The odds ratio (OR), 95% confidence interval (CI) and its respective *p* value were also calculated. Pearson’s correlation coefficients were calculated to evaluate the level of intrarater and interrater reliability. The interrater reliability was assessed by comparing the measurements between two of the authors. One of the authors examined a random sample of 20 patients. The intrarater reliability was assessed by comparing two separate measurements by a single author that were made 2 weeks apart from one another. The SPSS statistical software program version 19 was used to perform all statistical procedures.

## Results

A total of 93 patients who met the inclusion and exclusion criteria were enrolled in this study. Because 4 patients failed the VFS study, the data from 89 patients were analyzed. These patients included 71 males and 18 females with a mean age of 59.31±11.46 years. The median time from stroke onset to VFS study was 3 days (range 1–7). Thirty-five patients had supratentorial strokes, and fifteen patients had subtentorial strokes. Thirty-three patients had both supratentorial and subtentorial strokes, and six could not be localized. Twenty one patients were classified as aspiration (PAS ≧5)during the 5 ml diluted barium swallowing. Eleven patients were silent aspiration. One patient aspirated before swallowing while the others aspirated during swallowing. Eighteen patients aspirated less than 10% of 5ml barium and three patients aspirated 10–50% of 5ml barium. Table 1 summarized the baseline demographic and clinic information of the 89 patients. Table 2 demonstrated results from the univariate analysis comparing the VFS indices between patients with and without aspiration after stroke. Table 3 listed the indices which reached statistical significant of 0.05 level during the univariate analysis and results from the subsequent stepwise regression analysis. Because we identified a relationship between pharyngeal transit time and the two laryngeal elevation time (Pearson correlation coefficient *r* = 0.409 and 0.428, *p* = 0.000), the former was not entered into the regression analysis.

**Table 1:** Baseline demographic and clinical information and comparison of the VFS indices in patients using univariate analysis with PAS equal to or greater than 5 and less than 5.

Indices	PAS<5	PAS≧5	*p*
**age**	57±11	64±10	0.026
**gender**			0.158
**Male**	52	18	
**Female**	16	2	
**Location of stroke**			0.777
**supratentorial**	25	10	
**subtentorial**	11	4	
**both**	26	7	
**Abnormal lip closure**	7(58)	3(16)	0.403
**Decreased tongue movement and control**	23(41)	8(11)	0.409
**Barium residue in the pharyngeal**	47(21)	14(7)	0.516
**Invalid laryngeal elevation before a true swallowing**	5(63)	6(15)	0.018
**Abnormal epigglotis tilt**	17(51)	12(9)	0.007
**Latency of pharyngeal phase (s)**	0(-0.23–3.27)	0.13(-0.17–4.17)	0.04
**Duration from laryngeal resting position to maximum superior position(s)**	0.166(0.033–0.633)	0.233(0.133–0.700)	0.028
**DLVC (s)**	0.2(0.03–0.46)	0.2(0.13–0.76)	0.425
**Velocity for laryngeal elevated to maximal superior position (%/s)**	234(32.7–990)	167(81–300)	0.011
**Duration from laryngeal resting position to vestibule fully closed (s)**	0.1(0.03–0.56)	0.16(0.06–0.63)	0.021
**Velocity for laryngeal elevated to vestibule fully closed (%/s)**	2.66±1.15	1.95±0.82	0.012
**PTT (s)**	0.28(0.06–3.56)	0.41(0.2–5.16)	0.005
**Range of laryngeal elevated to maximal superior position (%)**	40.89±10.32	41.28±10.78	0.881
**Range of laryngeal elevated to vestibule fully closed position (%)**	35.25±11.23	34.84±10	0.883
**Duration of the UES opening(s)**	0.2(0.07–0.40)	0.16(0.17–0.50)	0.339

Normally distributed indices are indicated by the means±standard deviation. Indices with a non-normal distribution are indicated with the medians (min-max). DLVC: duration of laryngeal vestibule closure; PTT: pharyngeal transit time; UES: upper esophageal sphincter.

**Table 2:** Results from the logistic regression analysis of VFS indices identified from previous univariate analysis and their association with PAS equal to or greater than 5, including velocity and duration for laryngeal elevated to position where laryngeal vestibule is fully closed.

Measurements	B	OR	95% CI	*p* value
**Age**	0.072	1.074	1.014–1.138	0.015
**Duration from laryngeal resting position to vestibule fully closed(s)**	-0.06	0.942	0.749–1.185	0.609
**Velocity for laryngeal elevated to vestibule fully closed(%/s)**	-0.007	0.993	0.987–1.0000	0.046
**Latency of the pharyngeal phase (s)**	-0.045	0.956	0.371–2.461	0.926
**Abnormal epiglottis tilt**	1.39	4.015	1.2–13.437	0.024
**Invalid laryngeal elevation before a true swallowing**	1.482	4.4	0.697–27.78	0.115

OR indicated odd ratio. CI indicated confidence intervals.

**Table 3:** Results from the logistic regression analysis of VFS indices identified from previous univariate analysis and their association with PAS equal to or greater than 5, including the velocity and duration for the laryngeal elevated to maximal superior position.

Measurements	B	OR	95% CI	*p* value
**Age**	0.067	1.070	1.011–1.132	0.019
**Duration from laryngeal resting position to maximum superior position (s)**	-2.887	0.056	0–41.141	0.392
**Velocity for laryngeal elevated to maximal superior position (%/s)**	-0.009	0.991	0.981–1.000	0.059
**Latency of the pharyngeal phase (s)**	0.023	1.023	0.404–2.59	0.961
**Abnormal epiglottis tilt**	1.041	2.831	0.859–9.334	0.087
**Invalid elevation of laryngeal before a true swallowing**	1.502	4.493	0.741–27.249	0.102

OR indicated odd ratio. CI indicated confidence intervals.

Because the duration of laryngeal elevation from resting position to maximum superior elevation shows correlation with the duration from resting position to the position where the laryngeal vestibule is fully closed (pearson r = 0.853, p = 0.000), and the velocity of laryngeal elevation to maximal superior position and to its fully closed position, relatively, also has correlation (pearson r = 0.946, p = 0.000), therefore only one group of duration and velocity are subsituted in the equation when using logistic regression analysis. Also, as the pharyngeal transition time has correlation with the duration of laryngeal elevation either to maxium superior elevation or to its fully closed position (pearson r = 0.409 and 0.428, p = 0.000), thus PTT was eliminated from equation subsitution. Table 2 summarized the results of velocity and duration for laryngeal elevated to the position where laryngeal vestibule is fully closed. Table 3 summarized the results of veloscity and duration for laryngeal elevated to maximum superior position through logistics regression analysis.

The Pearson’s coefficients for interrater reliability for the location of the larynx at rest and at its highest level, the duration of larynx closure and the time required for the larynx to move from the rest stature to maximum elevation were 0.980, 0.973, 0.997 and 0.992, respectively, and those for intrarater reliability were 0.975, 0.990, 0.984 and 0.994, respectively.

## Discussion

In this study, penetration and aspiration was assessed through the PAS scale. In coherence with traditional concepts, a score of 5 and above is regarded as aspiration, and a score of less than 5 indicate no aspiration. In univariate analysis, the study found that for acute ischemic stroke patients with dysphagia, indices that associated with aspiration included age, invalid laryngeal elevation before a true swallowing, abnormal epiglottis tilt, prolonged laryngeal elevation time, prolonged pharyngeal transit time, latency of pharyngeal phase, and decreased laryngeal elevation velocity. Laryngeal elevation duration and velocity each has two parameters: laryngeal elevation from the resting position to maximal superior position and from the resting position to its fully closed position, relatively. Because the laryngeal vestibule is not yet fully closed, therefore duration and velocity of laryngeal elevation from resting position to its fully closed position have more significance in determining aspiration. Regression analysis findings showed after adjusting other indices involved, the velocity of which the laryngeal elevates to its maximal superior position has a tendency of predicting aspiration (p = 0.059, OR 0.991, 95%CI 0.981–1.000). The velocity of which the laryngeal elevates to its fully closed position can directly predict aspiration (p = 0.046, OR 0.993, 95%CI 0.987–1.000).

In the many indices associated with aspiration, the change of laryngeal elevation biomechanics is one of the more significant indices for prediction. Laryngeal elevation can facilitate the closure of the vestibule, repositioning of the larynx under the tongue base, and opening the UES [22]and is a core component of the airway protection from the aspiration. Normal swallowing involves hyolaryngeal elevation with precise timing to aid in airway protection [13]. physiological measures or kinematic measures of laryngeal elevation could reflect the mechanism of swallowing function impairment and then impact swallowing treatment decision[23]. Little research has been performed to quantify laryngeal elevation velocity and examine the relationship between the laryngeal elevation velocity and aspiration. Our study measured the laryngeal elevation velocity using VFS in a cohort of patients with acute ischemic strokes. There was a statistically significant difference in the laryngeal elevation velocity amongst patients with PAS score equal to or greater than 5 and less than 5 using univariate analysis (elevation to maximum superior position, *p* = 0.011; elevation to vestibule fully closed, *p* = 0.012). Especially after correcting confounding factors, logistic regression analysis indicates that laryngeal elevation velocity before the laryngeal is fully closed has more significance for aspiration prediction than that at its maximal superior position (*p* = 0.046 vs 0.059). Previous study identified that faster hyoid peak velocities contribute to an earlier LVC [18], and this phenomenon may also be true for laryngeal elevation and its implications for aspiration risk. Slowed laryngeal elevation velocity prolong the dwell time of the bolus in the pharynx while the laryngeal entrance is opening, thereby increasing the risk of aspiration. Morten et al found that bolus dwelling time in the pharyngeal was significantly longer in patients with aspiration than those without and longer bolus dwelling time in the pharynx while the laryngeal vestibule is not closed was correlated with an increased risk of aspiration[6].

There was no difference observed in the range of laryngeal superior elevation between aspiration and non-aspiration stroke patients in this study. Because there is a close correlation between excursion of the larynx and the hyoid bone [12], previous studies mainly focused on hyoid elevation could reflex larynx function. Previous study showed hyoid elevation range didn’t different with control subjects in stroke patients [24]. Kim et al found in their study that there were statistically insignificant differences in vertical hyoid excursion between aspirators and nonaspirators [25]. In the other hand, there were also previous studies showed reduced hyolaryngeal elevation is associated with aspiration. Reduced vertical excursion of the hyolaryngeal complex may lead to incomplete airway closure with an associated risk of aspiration [26]. Perlman et al. showed a significant correlation between reduced hyoid excursion and penetration/aspiration in their study, but reduced laryngeal excursion showed no predictive value for aspiration[17]. Kuhl et al. revealed in their study that the mean laryngeal elevation was reduced in neurogenic dysphagia patients [14]. Reduced hyoid elevation has been found to be associated with impaired swallowing and an increased risk of aspiration [15]. Swallowing therapy were shown to improve superior excursion of hyoid and larynx significantly in previous studies [16]. However, these studies included patients with heterogeneous diseases. This present study suggests that in stroke patients the velocity of laryngeal kinematics may be more important for LVC than the range of elevation [13].

In our study, age, abnormal epiglottic tilt,delayed pharyngeal phase, and invalid laryngeal elevation were identified as risk factors for aspiration during the univariate analysis. Age is a significant risk factor for aspiration especially with increased risk in individuals over 80 years[6]. As the age increases, the risk of aspiration increases gradually. In this study, patients with aspiration were more older than those who without aspiration (64±10 vs 57±11, p = 0.026). Epiglottic tilt is one of the airway’s protective mechanisms during pharyngeal swallowing [19]. Deviant epiglottis function may increase the risk of aspiration for four times than normal epiglottis function[27]. In our study, abnormal epiglottis tilt was associated with aspiration and independent predict aspiration after adjusted the other factors. Previous research has demonstrated that delayed pharyngeal phase is associated with increased aspiration risk [28]. Kahrilas et al demonstrated that delayed laryngeal elevation was associated with penetration and aspiration [19]. A delayed initiation of laryngeal closure from the time when the bolus enters the pharynx is a significant indicator of the overall risk of aspiration [9]. Invalid laryngeal elevation may indicate the insufficient muscle power to complete the elevation of laryngeal. Patients may try the best to elevate the laryngeal for several times and then complete a true swallowing. During the repeated invalid laryngeal elevation, bolus entered into pharyngeal and may spill into the opening laryngeal vestibule and pass under the vocal folds. The logistic regression analysis showed that only abnormal epiglottis tilt was still an indicator of aspiration after adjusted the effects of these confounding factors on aspiration in this study.

Our study revealed a positive correlation between laryngeal elevation time and velocity and the PTT (p = 0.005). This means a prolonged pharyngeal transition of the bolus can increase the risk of aspiration in patients with dysphagia. Previous studies have demonstrated that PTTs were prolonged in aspirating stroke patients when compared with the non-aspirating stroke patients, suggesting that a prolonged PTT was an indicator of aspiration risk in the post-stroke patients [10, 28].

The neural inpulse or inappropriate conductivity to or from the central nervous system (CNS) may indirectly affect healthy peripheral striated muscular system. This may be the same for the muscles involved in hyolaryngeal elevation [29]. A variety of neurologic diseases can affect the swallowing mechanism by impairing sensory and motor impulse trnsmissions and/or integration in the CNS [19, 29]. There are several muscles, such as the stylohyoid, stylopharyngeus, palatopharyngeus, salpingopharyngeus, mylohyoid, anterior belly of the digastric, hyoglossus and geniohyoid that are involved in the elevation of the hyolaryngeal complex [29]. Stroke patients with dysphagia may have impaired velar and hyolaryngeal elevation together with varying degrees of central laryngeal nerve paralysis [29]. Decreased elevation velocity of the larynx may be indicative of slow contraction of the suprahyoid muscles associated with abnormal muscle strength and decreased contraction velocity [30]. Therefore targeted dysphagia therapy should be aimed towards improving the laryngeal elevation velocity to reduce aspiration. Further elucidation of the mechanisms of swallowing may enhance the understanding of how swallowing is controlled by the CNS and thus improve current therapeutic methods or enable the development of new methods.

Measures of structural displacement during the swallowing of thin liquids should be corrected for variations in participant height [26]. It is inappropriate to compare structural displacement to references expressed in millimeters [26]. A wide range of superior laryngeal excursions have been documented across studies regarding the absolute distance [31]. Considering the radiologic amplification and differences in neck length among different individuals, our study used the percentage of laryngeal elevation in reference to individual neck length, rather than the actual distance in millimeters. Therefore, the analysis lacks comparability to other studies involving laryneal elevation [32–34].

The present study had several limitations. Firstly, this study was a single center cohort study with possible hospital bias. Secondly, patients with severe condition were excluded from this study, which may lead to selection bias. Thirdly, operators performed the measurements of the VFS study were authors involved in the study which may be a source of potential bias. Lastly, this study did not exam all the possible risk factors of aspiration such as reduced oral control function. Therefore further studies are needed to assess the laryngeal elevation velocity as a predictor for aspiration in stroke patients and validate our findings.

## Conclusions

Aspiration pneumonia is associated with aspiration in dysphagic acute stroke patients and is the leading cause of death following the initial stroke injury. Laryngeal elevation is an important protective mechanism from aspiration during the swallowing reflex. Studies have demonstrated that reduced hyolaryngeal elevation is indicative of impaired swallowing. However, few studies have focused on the effect of laryngeal elevation velocity on aspiration. In this study, reduced laryngeal elevation velocity was found to be a predictor of aspiration after adjusting for other swallowing parameters associated with aspiration. Slowed laryngeal elevation may be associated with decreased contraction velocity in the hyolaryngeal elevation muscles. Therapeutic methods aimed at improving larynx elevation velocity may decrease aspiration risk and incidences of aspiration pneumonia in patients with acute ischemic stroke and thus improve patients outcome.

## Supporting Information

S1 Database This is the database. (RAR)

S1 Fig This is the figure quality proof. (PDF)
