# Supplementary material for: Laryngeal Elevation Velocity and Aspiration in Acute Ischemic Stroke Patients
Source: PLoS One. 2016 Sep 1;11(9):e0162257. doi: 10.1371/journal.pone.0162257 (PMC5008618; doi:10.1371/journal.pone.0162257)
Supplement: S1 Fig — (PDF) [file pone.0162257.s002.pdf]

PLOS

Figure file quality report: 2016-07-19				
Original Filename	PACE Filename	Status	Error Detail(s)	PACE Adjustments
DRPSRS1W_Figure1	Fig1.tif	✓	• No Error	
